# Chronic Tophaceous Gout Masking Methicillin-Sensitive Staphylococcus aureus Bacteremia

**DOI:** 10.7759/cureus.109865

**Published:** 2026-05-29

**Authors:** Guy Tobias

**Affiliations:** 1 Medicine, York Central Hospital, Vaughan, CAN

**Keywords:** discitis, endocarditis, gout flare, infective endocarditis, sepsis, vertebral discitis

## Abstract

A 91-year-old woman with chronic tophaceous gout presented with worsening left foot pain, swelling, and purulent drainage after several months of presumed gout flares treated with prednisone and antibiotics. At presentation, she was septic with leukocytosis, elevated inflammatory markers, and imaging consistent with first metatarsophalangeal (MTP) osteomyelitis. Blood cultures grew methicillin-sensitive *Staphylococcus aureus* (MSSA). Further imaging demonstrated T12/L1 discitis and lumbar epidural phlegmon/abscess extending from L3/L4 to L5. Echocardiography showed a small filamentous structure on the mitral valve where vegetation could not be excluded. This case showcases diagnostic uncertainty when infection mimics gout and the need for early reassessment when symptoms fail to respond to conventional treatments.

## Introduction

Gout and soft tissue or musculoskeletal infections can share overlapping symptoms including pain, erythema, swelling, elevated inflammatory markers, and occasionally fever. Chronic tophaceous gout is characterized by the deposition of monosodium urate crystals in soft tissues which may lead to skin breakdown and can be a portal for infection. It may present with erythema, pain, discharge, and fever, which can confound an infectious process. It may be difficult to distinguish tophaceous drainage from purulent discharge [1].

Methicillin-sensitive *Staphylococcus aureus *(MSSA) bacteremia is associated with metastatic infection including vertebral osteomyelitis, epidural abscess, and infective endocarditis. Early recognition is essential due to high morbidity and mortality [2,3].

## Case presentation

**Figure 1:**
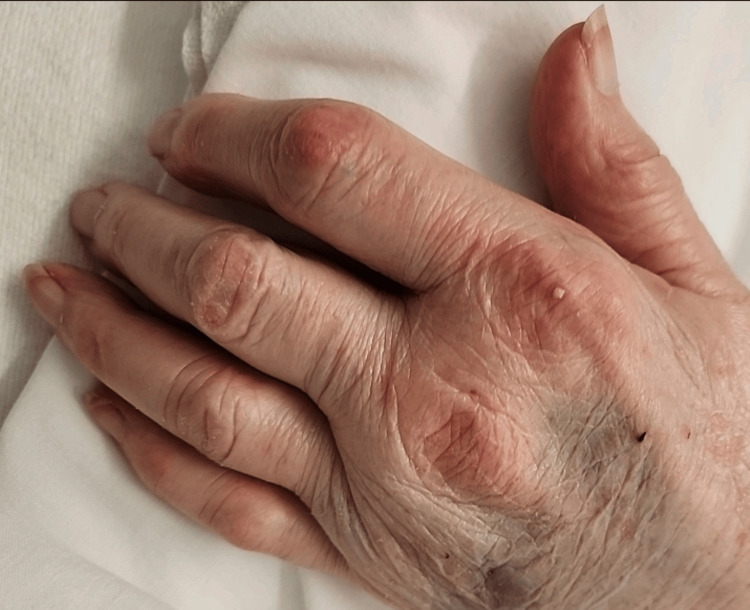
Left hand: tophi Chronic tophaceous gout with deformity of interphalangeal and metacarpophalangeal joints

A 91-year-old woman with chronic tophaceous gout affecting the hands and feet presented with progressive left foot swelling, erythema, pain, and presumed purulent drainage (Figure 1). She had recently relocated to a retirement residence and was ambulating with a walker for gait assistance.

For several months, she experienced recurrent episodes of suspected gout flares and cellulitis requiring medical attention and treatment with antibiotic courses including amoxicillin-clavulanate and prednisone 20 mg daily. She was not receiving urate-lowering therapy such as allopurinol. An outpatient radiograph obtained prior to admission demonstrated severe soft tissue swelling and destruction of the first metatarsophalangeal (MTP) joint in keeping with severe tophaceous gout versus osteomyelitis.

**Figure 2:**
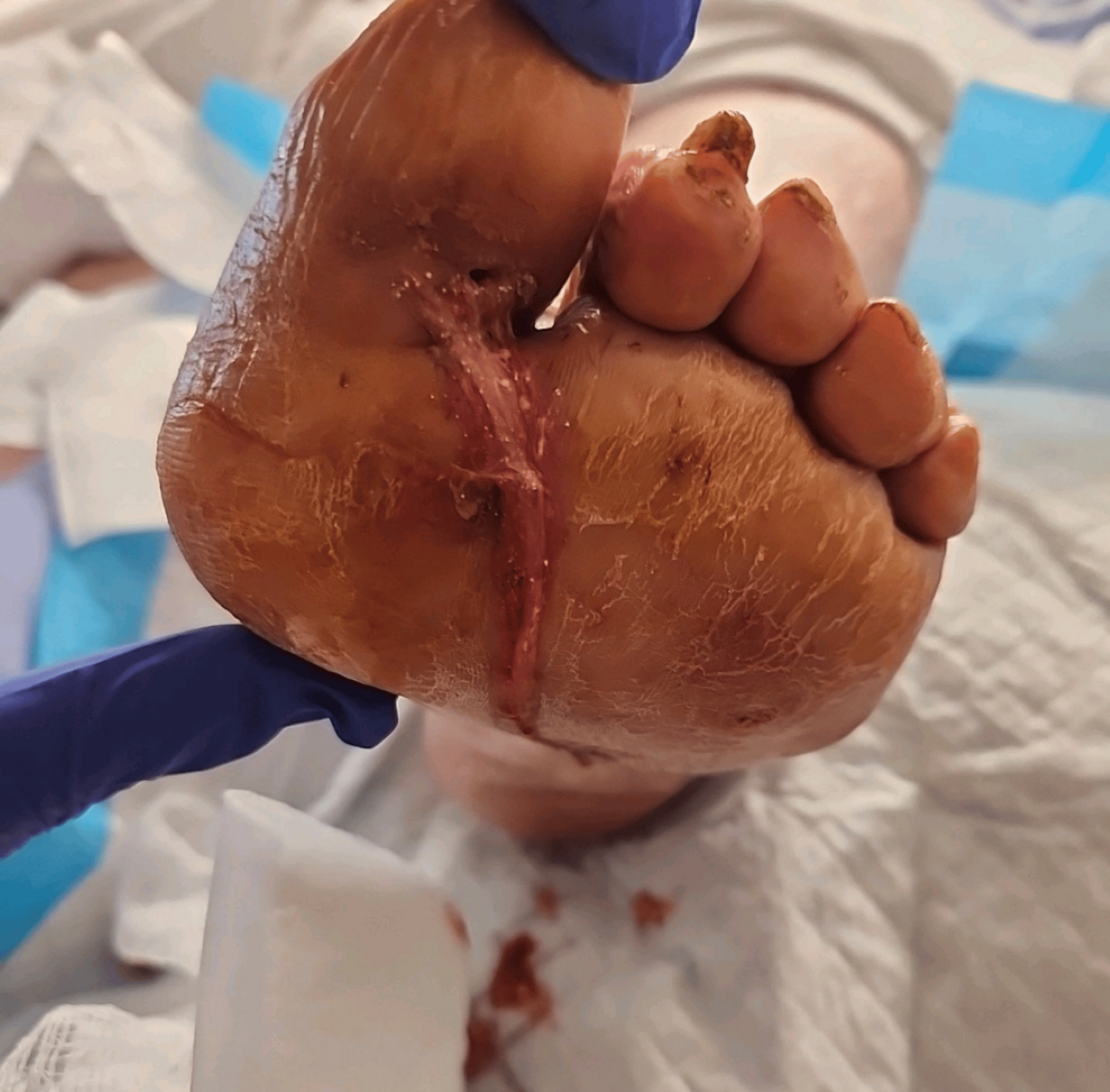
Left foot (plantar view) Plantar ulceration with draining tophaceous material at the base of the left great toe

**Figure 3:**
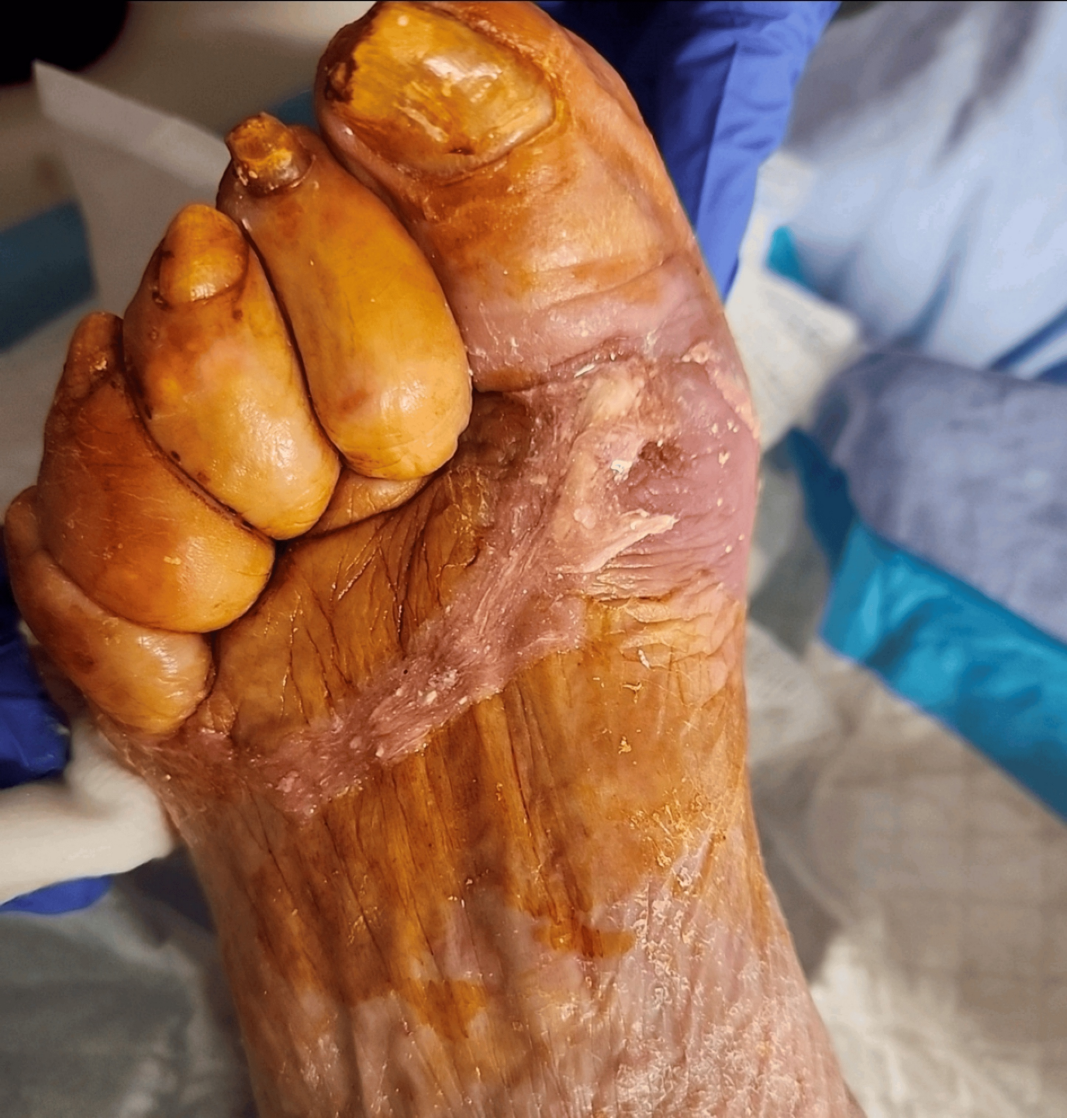
Left foot (dorsal view) Extensive tophaceous gout with skin breakdown and sinus tract formation

Her symptoms progressed with the development of presumed purulent drainage between the first and second toes, fever, and inability to ambulate (Figure 2). In the hospital, discharge from the dorsal and plantar aspects of the first MTP appeared chalky and consistent with known gout (Figure 3). The open wounds could have been secondarily infected and were considered a likely source of infection.

On presentation, she was febrile (38.5°C), tachycardic, and borderline hypotensive.

Laboratory investigations demonstrated marked leukocytosis with a white blood cell (WBC) count of 33.2×10⁹/L, C-reactive protein (CRP) of >240 mg/L, lactate of 3.5 mmol/L, and creatinine of 183 µmol/L from a baseline of approximately 140 µmol/L, consistent with severe inflammatory response, sepsis, and acute kidney injury on chronic renal impairment (Table 1).

**Table 1:** Laboratory results

Test	Result	Reference range (typical adult)	Interpretation
White blood cell (WBC) count	33.2×10⁹/L	4.0-11.0×10⁹/L	Marked leukocytosis; concerning for severe infection/inflammation, physiologic stress, or hematologic disorder
C-reactive protein (CRP)	>240 mg/L	<5 mg/L	Extremely elevated inflammatory marker; commonly associated with severe bacterial infection or major inflammatory process
Lactate	3.5 mmol/L	0.5-2.0 mmol/L	Elevated; may indicate tissue hypoperfusion, sepsis, or severe physiologic stress
Creatinine	183 µmol/L (baseline ~140 µmol/L)	~60-110 µmol/L (lab-dependent)	Elevated; acute worsening from baseline, suggesting acute kidney injury on chronic renal impairment

Repeat foot radiographs showed destructive changes of the first MTP joint consistent with osteomyelitis.

Blood cultures grew MSSA, and she was treated for MSSA bacteremia with cefazolin [2].

During admission, she developed worsening back pain, decreased mobility, and urinary retention. Magnetic resonance imaging (MRI) of the lumbar spine demonstrated epidural signal abnormality at L3/L4 extending to mid-L5, a rim-enhancing epidural phlegmon/abscess, and facet joint and interspinous involvement with marrow edema. Findings were consistent with discitis and epidural abscess related to MSSA bacteremia [4].

Transthoracic echocardiography demonstrated mildly calcified mitral valve leaflets and a 2×3 mm filamentous strand on the atrial surface of the anterior mitral leaflet, representing degenerative change versus vegetation, and infective endocarditis could not be excluded [5].

She was treated with prednisone for gout flare management and was initiated on allopurinol. Internal medicine and infectious disease services followed her throughout the hospitalization.

## Discussion

This case illustrates how severe chronic tophaceous gout can mask a serious metastatic infection including sepsis, osteomyelitis, discitis, epidural abscess, and possible infective endocarditis as seen in this patient. Ulcerated tophi with chalky drainage can mimic chronic gout-related inflammation while also serving as a portal of entry for invasive infection.

Several findings supported an infectious process in this patient, including recurrent symptoms despite treatment, persistent pain and drainage from the first MTP joint, markedly elevated inflammatory markers despite gout-directed therapy, imaging findings with an infectious process included in the differential, and progressive systemic symptoms despite antibiotics and corticosteroids.

The coexistence of gout and infection created diagnostic uncertainty, as both may present with erythema, swelling, leukocytosis, and fever. Chronic kidney disease, repeated corticosteroid exposure, and delayed urate-lowering therapy likely contributed to the progression of both uncontrolled gout and occult infection. The combination of ulcerated first MTP tophi complicated by MSSA bacteremia with metastatic spinal infection appears rarely described and adds to the limited literature describing ulcerated gout as a source of invasive systemic infection.

MSSA bacteremia requires evaluation for metastatic infection. Vertebral osteomyelitis and epidural abscess may initially present with nonspecific back pain before neurologic deficits develop. She later developed worsening mobility and urinary retention requiring Foley catheterization, prompting spinal imaging that demonstrated discitis and epidural abscess [3,4]. Holland et al. emphasized that *Staphylococcus aureus* bacteremia carries substantial risk for metastatic complications including vertebral infection and endocarditis [2].

Early detection is critical, as delayed diagnosis is associated with substantial mortality. In patients older than 85 years, all-cause 30-day mortality can exceed 50% [6].

Echocardiography was also suspicious for infective endocarditis. Transesophageal echocardiography was not performed. The patient was treated with at least six weeks of parenteral cefazolin, and follow-up transthoracic echocardiography was planned to reassess the possible vegetation [5].

Published reports remain limited; Filanovsky et al. described ulcerated tophaceous gout as an uncommon complication of poorly controlled hyperuricemia, with only a small number of reported cases involving secondary infection or osteomyelitis [7].

## Conclusions

Recurrent, non-resolving, or worsening gout flares should prompt evaluation for infection. At the very least, it should prompt re-assessment of treatment options and consideration of underlying infection. In this case, presumed gout flares masked MSSA sepsis complicated by discitis, epidural abscess, and possible infective endocarditis. Early recognition, appropriate investigations, and early intervention are essential for progressions, complications, and positive outcomes.
